# Z60.5/(En)Coded

**DOI:** 10.1089/heq.2023.0033

**Published:** 2023-11-30

**Authors:** Ryan J. Petteway

**Affiliations:** Community Health, OHSU-PSU School of Public Health, Portland State University, Portland, Oregon, USA.

their children wear our jerseys

[they race adjust our brains]^1^

they think we need attitude adjustment

they settle for statistical adjustment^2^

they can't control for justice

they can't control our edges

our knees (*their* knees)

[they race adjust our lungs]^3–6^

our hips (*their* hips)

our fists

our mouths

our lips on their daughters

their daughters on *TikTok*

their sons on sigs, glocks

their silence __________

_________

our matter

our matters

our mattering

[they race adjust our pain]^7–9^

they can't control our anger

they think they know our rage

they settle

they: settler

they: eyes

our streets

our parks

our schools

our playgrounds

our grounds

our play

our joy unfiltered

[they race adjust our blood]^9–14^

our names they don't pronounce

they squiggly red line our names

they redlined our streets

they raced to scrub our names

from rivers

from mountains

from lakes

from stars

from murals

[they race adjust our homes]^15–17^

they search to find our magic

our glow

our radiance

they say they do not see

they look right through us

[they race adjust our bones]^18^

their hands held the compass

the maps

the pens

the gavels

the glocks

the signs in Little Rock –

their children love our dances

their faces look the same

their cameras track our faces

their mothers don't feel safe

our mothers: open casket

our faces make front page

our fathers fit descriptions

our daughters go unfound

our sisters ain't Serena

[they race adjust our births]^19–21^

they say they ally

they say they here

they say they hear

they said that last year

they say they listen

to Kendrick

to Nina

to Toni

to Toni

to Audre

to James

to god

they love a soundtrack

they love to sound track

[they race adjust our cars?]^22^

they good intention

they good intention real good

they well mean

they racist bone-free

[*they be tryin’ so hard*]^23–25^

their hands are not clean

their hands wrote the code

our hands get cuffed in labor

our hands cannot get soap

our lungs cannot get oxygen

their algorithms rope^†^

their software comes with “bootstrap”

and that's all you need to know

## Notes

^†^
“Rope” is a technical term for a type of data structure, usually a (binary) “tree” data structure… Go figure.
